# On The Problem of Defending Basic Equality: Natural Law and The Substance View

**DOI:** 10.1093/jmp/jhad030

**Published:** 2023-07-05

**Authors:** Henrik Friberg-Fernros

**Affiliations:** University of Gothenburg, Gothenburg, Sweden

**Keywords:** *basic equality*, *justification*, *natural law*, *rational agency*, *substance view*

## Abstract

While most theorists agree with the claim that human beings have high and equal moral standing, there are strong disagreements about how to justify this claim. These disagreements arise because there are different ways of managing the difficulty of finding a basis for this claim, which is sufficiently substantial to do this justifying work, but not vary in degree in order to not give rise to inequality of moral considerability. The aim of this paper is to review previous attempts to address this difficulty and to demonstrate why they fail and then to defend another way of dealing with this challenge by applying two views: the substance view on the human person and the natural-law account of morality. My claim is that this approach has comparative advantages because it provides a binary and a normatively significant basis of justification for equality without being implausibly inclusive.

## I. INTRODUCTION

While most theorists subscribe to the claim that human beings have high moral standing and basic equal rights, there are strong disagreements about how this should be justified.^1^ These disagreements arise because there are different ways of managing a difficulty that resists attempts to justify this kind of high and equal moral standing. The philosopher Richard Arneson spells out the difficulty of finding a basis for this position in the following way:

Either the proposed basis will turn out to vary by degree, and will give rise to inequality of moral considerability, or the proposed basis will turn out to be one that applies in all-or-nothing fashion, and then it will turn out that the basis proposed as justifying equal moral considerability is too flimsy or insubstantial to do this justifying work. (2015, 42)

I think that this gloomy conclusion is premature. Not primarily because I believe that some of these previous attempts have been successful; rather, I tend to agree with Arneson in his conclusion that they do not seem to be acceptable. However, I believe that the resources of two particular views, which at least prima facie seem to have good potential to justify the position that human beings have a high and equal moral standing, have not yet been sufficiently explored in this context. The views I am considering are the substance view on the human person and the natural-law account of morality. According to the substance view, each human being has high and equal moral standing due to her rational nature. As one either has or does not have a rational nature, the substance view consequently provides a binary criterion for determining the moral standing of human beings. By applying the natural-law account of morality, the aim is to provide a normative framework that can determine what kind of obligations this moral standing generates.

While it is quite common that these two positions are simultaneously held and defended, I believe that the combination of these views is especially adequate regarding this discussion. The substance view implies a quite inclusive criterion for determining the moral standing of human beings, which, prima facie, might be considered to generate implausibly far-reaching consequences.^2^ However, combined with the natural-law account of morality—according to which universally moral principles are usually formulated negatively and consequently in a less far-reaching way—this inclusiveness can be managed by mitigating the alleged implausibility of the consequences of this inclusiveness. I defend an inclusive but quite minimalistic approach to how one can justify the high and equal moral standing of humans.

While I think that the substance view and the natural-law account of morality provide a promising way of justifying the high and equal moral standing of human beings, I do not claim to defend this approach conclusively. Rather, I aim to demonstrate that this approach has some comparative advantages over other attempts, and that some initial worries about its plausibility can be met. In other words: I aim to argue for the rationale of considering this approach to respond to the challenge of justifying why human beings have high and equal moral standing.

This paper unfolds in the following way. First, I demonstrate the problems with previous attempts to tackle the difficulties of defending a basis for the high and equal moral standing of human beings. Second, I lay out the response from the substance view on the human person and the natural-law account of morality, which I claim has comparative advantages. I then respond to some objections before I finish by making some concluding remarks.

## II. THE CLAIM AND WHY THERE ARE PROBLEMS WITH RECENT ATTEMPTS TO DEFEND IT

The claim to be defended is that human beings have high and equal moral standing (Sher, 2015, 17). This claim (hereafter the claim) does not imply that people should always be treated equally; defenders of the claim are, of course, not obligated to hold the view that perpetrators should be treated in the same way as their victims. The way people choose to act may justify that we differentiate between people. Rather, the claim is rooted in principles that assume that people—under capacities we have or merely the way we are constituted—have equal moral standing (Sher, 2015, 16). Consequently, existence as such or capacities are usually considered as possible candidates for the justificatory ground for the claim, while actions are not.

However, according to the claim, not only do people have equal moral standing; but also *high* moral standing: theoretically, mere equality does not exclude that people should be treated with very low or even no moral standing at all as long as such a “principle” is applied equally, but that is usually not what is meant by this claim.

Once we have established the grounds for the claim, the question naturally arises about why the nature of human beings requires that we have high and equal moral standing. Why are human beings that special? Merely the fact that we are human seems unnecessary (and circular); if we someday discover extraterrestrial life or an animal with the same rational capacities as human beings, it would seem arbitrary to treat them unequally simply because they are not human beings. The justificatory basis of the claim must be grounded on something we are or have as human beings—which principally could be shared by other species rather than merely belonging to the human species (Sher, 2015, 17). In other words: it must meet the Species Neutrality Requirement (Liao, 2010, 162).

The most promising candidates for such a justificatory basis of the claim are related to human rationality (Sher, 2015, 16; Waldron, 2015, 92). Human rational agency is described by Arneson as the ability to “identify available courses of action she might take, discern reasons for and against the options, weigh and assess the reasons she discerns, deliberate and make choices, carry out the action chosen, and do all this not simply for a single decision problem at a time but with respect to long-term plans of action” (2015, 33–4). However, once the relevance of the rational agency as a justificatory basis for the claim is acknowledged, worries about the scalar nature of human agency are raised. Surely, rational human agency—manifested in terms of the capabilities just mentioned—varies between human beings. If this capability is invoked to justify our higher moral standing compared to other animals, then it follows, one might object, that these differences between human beings should also matter.

Furthermore, if such differences between human beings should matter, then this quality cannot be invoked to justify the claim about *equal* moral status between human beings. Rather, this implies that more rational people should have a higher moral status than less rational people. We can call this worry the objection from continuity (Christiano, 2015, 60). The question is whether we can identify and defend such a binary property as a ground for the moral standing of human beings. Below I review two recent attempts by George Sher (2015) and Thomas Christiano (2015).^3^ While I think that their views have merits, I argue that their defenses fail in the end. Instead, we should ground the claim on the substance view and natural law.

## III. GEORGE SHER´S AND THOMAS CHRISTIANO’S ATTEMPTS TO DEFEND THE CLAIM

Drawing on Bernard Williams’ earlier work, the philosopher George Sher argues that the mere fact that we as human beings have a subjective point of view—“organized around evolving sets of reason-based aims”—might constitute such a binary property (2015, 39):

If the reason we are moral equals is simply that each of us has (is?) a subjectivity of a certain sort—that each occupies a point of view from which the world appears a certain way, certain things appear to matter, and certain courses of action appear to be open—then any variations in the contents of our beliefs and aims, and in the capacities that gave rise to these, will simply drop out as irrelevant. (2015, 36)

Further, Sher insists that this property—that we have a subjective point of view—is a binary property that avoids problems with continuity:

A person who is semi-conscious, or who is fully awake but intellectually primitive or very stupid, has no less complete a subjectivity than a hyper-alert, hypersophisticated genius. This all-or-nothing aspect of our subjectivity is crucial to my argument, because it implies that the moral standing to which the subjectivities of different individuals give rise is all-or-nothing as well. (2015, 42)

The question is whether this is correct. Can having a subjective point of view differ in degrees? Now, it is clear that there are variations of the phenomenon for which Sher is arguing. Consider first how Sher describes the assumption that underlies this property:

These include, but are far from exhausted by, the assumptions that the world is temporally as well as spatially ordered, that the person himself is an embodied subject who has existed in the past and will exist for at least some time in the future, that various courses of action are open to him, that the world gives him reason to do some things and refrain from doing others, and that he is, within limits, capable both of finding out what reasons he has and of acting on them. (2017, 38)

Taking this description as a point of departure, the philosopher Stan Husi (2017, 398) asks us to consider a version of subjectivity that does not contain the last feature, reasons-sensitivity. That would certainly be a different version from the kind of subjectivity that includes reasons-sensitivity, but still a kind of subjectivity. Likewise, it seems reasonable to say that lacking reasons-sensitivity is not only a different version of subjectivity, but also a less rich version. Consequently, Husi argues, subjectivity is a matter of degrees, which means that the objection from continuity can also be raised against this property.

One way to respond to this objection is to claim that Sher’s assumptions are necessary for the relevant kind of subjectivity. That argument means that if one lacks one of these features—such as reasons-sensitivity—then one also lacks the kind of subjectivity that constitutes the justificatory basis for the claim that human beings have high and equal moral standing.

However, such an interpretation is probably beyond what Sher meant since he explicitly claims that the assumptions mentioned above are not exhaustive (2015, 38), which means that assumptions can be added to his list without changing the status of subjectivity. It seems, therefore, those specific assumptions are not necessarily constitutive elements of the relevant subjectivity. Nevertheless, one could of course argue for such a position anyway—even if Sher himself does not take this position—but, as Husi (2017, 398f) also demonstrates, just as it is possible to subtract assumptions from Sher’s list, it is also possible to add assumptions. The ability to see our fellow human beings as, for instance, “ends in themselves”—and consequently not use other human beings merely as a means to achieve a goal—does not appear on the list; however, a subjectivity that includes this aspect is arguably richer than a subjectivity that does not. This conclusion indicates that Sher’s proposal does not avoid the objection from continuity.

Another objection to Sher’s proposal is that actualized subjectivity might be too restrictive. While I believe that it is plausible to conclude that human subjectivity is relevant to human value somehow, I nevertheless reject the claim that actualized subjectivity is a necessary property for high and equal human value. To substantiate this claim, consider the following thought experiment. Twins, Alpha and Omega, are delivered within a 10-min interval. Now, let us make the admittedly quite bizarre assumption that the twins will later gain the relevant kind of subjectivity within the same interval in which they were delivered. Consequently, Alpha will gain full moral value 10 min before Omega. This means that if, for instance, their parents need to make a tragic choice between the survival of Alpha and Omega because of the lack of access to medicine only seconds before Omega would gain the right kind of subjectivity, such a choice would be between one entity that has full moral standing and one that does not. Thus, even if we expected Omega to have significantly greater chances for survival in such a situation, we would arguably be obligated to give the medicine to Alpha merely because he—having already gained the right kind of subjectivity—has full moral standing in contrast to Omega.

Moreover, on a more general level, by anesthetizing and keeping infants unconscious, we can prevent them from obtaining full moral status. That fact might justify not only infanticide but also other kinds of organ harvesting and live experimentation (Rodger, Blackshaw, and Miller, 2018). I believe that these implications are indeed implausible. Consequently, Sher’s proposal is problematic, because it is seemingly not binary, but it is also too restrictive.

Thomas Christiano proposes another way of grounding the claim. He contrasts Sher´s subjectivist account of human high and equal value with an account based on “impersonal worth idea” (Christiano, 2015, 58). According to this view, “the possession of reason endows a person with a kind of impersonal worth” that differentiates us from (at least most) non-human animals (Christiano, 2015, 58). While he willingly admits that this property is scalar in nature—and therefore allows for differentiation not only between human beings and non-human animals, but also between human beings—he argues that rationality as such is sufficient to justify a “non-comparative” norm. According to this norm—which, Christiano argues, does not allow for differentiation—humans are not allowed to be used merely as a means for other people’s purposes:

The scholastic idea is that inasmuch as human beings are rational beings, by which they mean that they are capable of running their own lives in accordance with values and principles they understand and can reach by means of reason, persons are not made merely for each other’s use. The idea here is that each person has a kind of original right against others. This right is initially a notion that is understood non-comparatively. (2015, 63)

Christiano further elaborates why this approach addresses the continuity objection effectively:

The reason is that the principle merely moves from a trait of a person to the idea that a person is not to be used merely for another’s purposes. This latter injunction has a non-comparative character, at least in its basic content. It does not admit of the idea that one may treat one person some of the time or in some respects as a means while others may never be treated as mere means. One simply looks at each rational being, and because of its character as rational and self-determining, it is fitting that it not be used merely as a means. (2015, 64)

I believe that this is a promising approach. Admittedly, as Christiano himself acknowledges, this approach merely states that one person may not be used “for the sake of the well-being of another and that one person’s well-being may not be sacrificed for the sake of others,” which some egalitarians might consider as too minimal.^4^ Nevertheless, this approach seems to address the continuity objection adequately. To not treat any rational human person merely as a means seems to be a binary norm without room for degrees. As Christiano notes, this norm does not admit “the idea that one may treat one person some of the time or in some respects as a means while others may never be treated as mere means” (2015, 64).

And this norm applies as long as the human person is rational—no matter to what extent one exhibits this capacity. The only human beings to whom this norm does not apply are those who lack this rational capacity; according to Christiano, these human beings “do not have the same status as the vast majority of human beings” (2015, 63).

By demanding actualized rationality, Christiano runs, however, into the same problems as Sher does when it comes to infants who have not realized their rational capacity. Applying the case above about the twins to Christiano’s approach—and thereby replacing subjectivity with rationality—he faces the challenge of explaining why Alpha, whose rationality will be realized some seconds before Omega’s, has full moral standing in contrast to Omega. But Christiano also faces challenges beyond that of human beings who lack rationality; for example, people with only partially realized rationality—such as human beings with cognitive disabilities—fall below his threshold. What about them? In an endnote, he addresses this challenge by noting that this approach does “not imply that we treat severely cognitively impaired human beings in the same way as animals” (2015, 75, endnote 9).

However, the claim that his approach does not *imply* such a treatment is a weak defense. The central question is whether it is *permissible at all* according to his approach to treat them as animals. And I cannot see why it would not be—even if we might grant that we are not *obligated* to treat them like animals. Suppose the actualization of rationality constitutes the threshold. In that case, it seems arbitrary to treat an animal with the same degree of realized rationality differently compared to a human being with the same level of rationality. Certainly, there might be external criteria that differentiate animals and cognitively disabled human beings. For example, human beings are generally more loved by human persons than animals are. Thereby, they might deserve better treatment than animals. However, such considerations are entirely contingent. If human persons loved an animal more, or if we all as persons were entirely indifferent to cognitively disabled persons, then it would be permissible to treat them worse than animals. For instance, rather than transplanting organs from animals to human beings, Christiano’s view seems to permit that we transplant organs from mentally disabled people to save human beings with fully actualized rationality.

I believe that these implications are implausible and fatal for a theory about human equality. And the problem is that Christiano’s threshold is merely based on actualized rationality and therefore is not sensitive to considerations about the nature of different beings. As I see it, while the animal and a cognitively disabled person in the example might have the same level of exercisable rationality, there is nevertheless one important difference: human beings have an intrinsic potentiality to actualize rationality in contrast to (at least most) animals. That fact, I think, should matter normatively. To illustrate the normative force of this aspect, consider the following situation. We have one serum unit that would make either a cognitively disabled human being or an animal rational. Intuitively, I think that most of us would have chosen to give the serum to the cognitively disabled person rather than the animal. And I think that this intuition is also sustained if we assume that the disabled person as well as the animal is isolated from their fellows—that is, our intuition does not rest on the fact that the animal would diverge in relation to its fellows in contrast to the human being. While we would add a capacity to the animal in conflict with its nature, we would restore something which was missing by giving the serum to the human being. And I think that this difference in nature explains this intuition, something which I develop in the next section, in which I defend an approach to human equality and value based on the substance view and natural-law morality.

## IV. THE SUBSTANCE VIEW AND NATURAL-LAW MORALITY AS A RESPONSE TO THE PROBLEM OF JUSTIFYING HUMAN EQUALITY

Despite significant differences between Sher´s and Christiano’s solutions to justify the Claim, both accounts share the view that the justificatory basis must be exercisable—either as a subjective point of view or as the possession of reason. I have argued that such a basis is too restrictive and less adequate in addressing the objection from continuity. In contrast, the justificatory basis for the claim of the substance view is human being’s rational nature, which is not an exercisable capacity. Rather, according to the substance view, human beings are valuable because they are beings with a rational nature. Having a nature constitutes an entity´s existence, which means that if an entity loses its nature, it ceases to exist (Lee, 2015, 112–3). The philosopher Christopher Kaczor calls this a “constitutive property”—a property of an entity necessary for its existence (2011, 113). Just as a triangle needs the sides to exist, human beings need a rational nature to exist. The philosopher Patrick Lee puts it in the following way:

… the criterion for full moral worth and possession of basic rights is not a capacity for conscious thought and choice which inheres in an entity, but being a certain kind of thing, that is, having a specific type of substantial nature. Thus, possession of full moral worth follows upon being a certain type of thing or substance, namely, a substance with a rational nature, despite the fact that some persons—have a greater intelligence or are morally superior—than others. Since basic rights are grounded in being a certain type of substance, it follows that just having such a substantial nature qualifies one as having full moral worth, basic rights, and equal personal dignity. (2004, 124)

Thus, this criterion is binary, which means that this view can meet the continuity objection.

This view is, moreover, more inclusive than those discussed above, since having a rational nature does not require the actualization of rationality. Rather, what is needed is an internal directedness to rationality (Lee, 2013, 243). One leading proponent of this view explains this concept in the following way:

Let me note, first, that human nature, or internal directedness toward the mature stage of a rational animal, is not located solely in the genes. Rather, the genetic-epigenetic state of the cells of a conceptus or embryo is a material cause, and having the right sort of genetic-epigenetic constitution is a necessary condition and an indication that what is present is a rational animal. (Lee, 2013, 244)^5^

Consequently, a human being is from the very beginning—biologically speaking—a rational animal with a rational nature, which means that human beings are valuable as long as they have a rational nature. Such a threshold is both inclusive and binary, which is a comparative advantage concerning the other views discussed above. Rather, the challenge for proponents of the substance view is the opposite: not only to justify why we should value an entity with the unrealized—while inherent—potentiality of rationality but also how this potentiality can constitute a basis for equality. As Christiano notes:

Only if a being has great worth can the duties we have to it and rights it possesses be of great normative weight. Moral principles often impose heavy burdens on practical agents. It is hard to see how these sacrifices can be justified without a sense that the beings toward whom they are owed are themselves of great worth. (2015, 60)

There are at least two difficult questions for proponents of the substance view to answer: First, how can one justify that an entity without any actualized rationality is valuable? And second, granting that this can be done, the even harder question remains to be answered about how one can justify that such an entity and a human being with actualized rationality are equal in any sense.

Some of my arguments against the previous views are, I think, relevant when addressing the first question about the value of human beings without actualized subjectivity and/or rationality. For instance, I believe it would be implausible that one would be obligated to give the medicine to Alpha even though Omega would have significantly greater chances to survive merely because he—having gained subjectivity and/or rationality 10 min before Omega—has full moral standing compared to Omega. This conclusion can be strengthened further by making the time difference even more marginal—like, for instance, 1 min. According to Sher´s and Christiano’s accounts, it is still mandatory to give the medicine to Alpha. In contrast, the substance view allows parents to choose to give the medicine to Omega, even though this view certainly does not imply that such a choice would be obligatory. I believe that this conclusion demonstrates the comparative advantages of this position over Sher’s and Christiano’s positions.

In a more general manner, the philosopher Patrick Lee launched the following thought experiment to illustrate the normative strength of ascribing value to entities without actualized rationality:

Let us suppose I have something seriously wrong with my brain, and unless I have brain surgery, I will die very soon. So, let us suppose that next week I will be admitted to a hospital, where I will undergo major brain surgery. But it is also virtually certain that one of the side effects of this surgery is that I will suffer complete amnesia—even to the point of losing all my linguistic abilities, abilities to walk, to eat with a fork, and so on. Now, of course, I wish I could avoid these side effects. I do not want to lose all my memories, personality traits, and so forth. After the surgery, I will not remember my wife, my children, any of my friends, or my deeply held beliefs. In a way, I will be starting all over. In fact, after the surgery, it will also take some time, say, several months, for me even to regain consciousness—I will gradually regain consciousness—but not the same content that I had before—and from there, I will have to gradually acquire knowledge, human relationships, and so on. (2013, 237)

When I am unconscious in this thought experiment, my position is similar to that of the embryo. Like the embryo, I will regain consciousness only after several months, which is a necessary condition for the actualization of rationality later on. However, it seems highly implausible to hold that I lack value as long as I am unconscious. To see that, let us make a further addition to the thought experiment and assume that the doctors can know when I will regain consciousness. Up to minutes (and even seconds), before I will regain consciousness, I will lack value according to both Sher’s and Christiano’s accounts, which means that I, just minutes (or seconds) before regaining consciousness, could be killed if that would be advantageous for others (if, for instance, my organs were needed). In contrast, in some minutes (or seconds) later, I would be protected from such actions as everybody else when I have regained consciousness. I find such a scenario as implausible and, consequently, comparatively advantageous, if an account of basic equality could block such implications.

Now the substance view provides a reason for doing that: I am the same substance during my unconscious time, and as a substance with a potential for rationality, I am valuable as long as I exist. And the same holds for the embryo, which will also be unconscious for a limited time before its capacity for consciousness and rationality will be actualized.

Certainly, in the thought experiment, I was once conscious, in contrast to the embryo. However, it is unclear why that should matter normatively. There is a discontinuity between the quality that once warranted my value—my previous consciousness and rationality—and my forthcoming consciousness and rationality. Therefore, to invoke my previous consciousness and rationality to justify why I now have a value during unconsciousness would be like making claims for benefits related to a certain position just because I once held such a position. The fact that I once held such a position—as a mayor, for instance—does not justify such claims if I have now lost this position. Normatively speaking, the embryo and I, therefore, seem to be in the same position. If we think that it would be impermissible to kill me, then it seems to follow that it would also be impermissible to kill an embryo.^6^ Granting that we intuitively agree that it would be wrong to kill me in Lee’s thought experiment above, then the substance view can explain this position while the competitors previously discussed cannot.

However, it is not sufficient to defend the plausibility of ascribing some kind of moral value to human beings simply because of their existence; I must also address the second and even harder question above, namely, how one can justify that an embryo and human being with actualized rationality are equal in any sense. To make sense of this claim, I believe that we must qualify the way proponents of the substance view should defend basic equality in a way that is not usually done. Such a revision is necessary because it seems very hard to deny that the actualization of rationality means that values are added. As the philosopher and one of the leading proponents of the substance view, David Oderberg concludes: “the presence of such things as reason, reflection, and self-consciousness [are] somehow crucially relevant to moral status….” (2000, 176)

To admit that the actualization of rationality adds value implies, as far as I can see, that we need to differentiate between a human being with and an embryo without actualized rationality in some way. But granting that we accept the intuition defended above about the impermissibility of killing me while I am unconscious, we cannot say that this differentiation means that we are permitted to destroy an embryo while an adult human being has a right to life. Rather, both the embryo and the adult human life have a right to life—that is, a negative right not to be killed. However, other rights might be differentiated due to this difference in value between entities with and without actualized rationality, respectively. Consider, for instance, a situation where you are forced to choose between saving a 10-year-old conscious child or an embryo. I believe all of us would choose to save the child; however, I also believe that we would save the conscious child if an unconscious 10-year-old child replaced the embryo. Consequently, we tend to value the mere actualization of consciousness and rationalization. Can we accommodate this with the substance view?

I think we can. Drawing on natural-law theory on morality, I think it is reasonable to assume that certain basic human goods are central to our well-being and should therefore guide our actions. Natural-law theorists argue that there is a plurality of such goods, and usually, they include phenomena such as life, knowledge, play, friendship, aesthetic experience, practical reasonableness, and religion. A common denominator for these goods is that it makes sense to realize these values merely for their own sake—and that it is wrong to act deliberately against these goods (Finnis, 2011).

Now, there are, of course, objections to this view. However, my aim here is not to defend this account of morality conclusively, but merely to demonstrate how it can complement the substance view to make sense of a kind of basic equality.^7^ To do this sensemaking, I propose that we distinguish between acting deliberately against a basic good—for example, a human life—and omissions to promote basic goods. While the former is impermissible, the latter is less clear-cut. Sometimes we are justified in not promoting basic goods while we, according to natural law, are never justified in deliberately acting against basic goods.^8^ How can we justify this sharp differentiation between the permissibility of not promoting basic goods and the impermissibility of deliberately acting against basic goods? It is clearly outside the scope of this paper to defend this distinction. Rather, I here draw on the general observation that undergirds this distinction within natural-law theory according to which “the diversity of human goods and the ways of achieving goods” implies that we can more often “state in universal terms what human beings ought not to do than what they ought to do” (Wolfe, 2006, 175). That, of course, does not mean that we merely are obligated to refrain from acting and never obligated actually to perform actions. For instance, faced with the choice of either saving the embryo or the child, we are obligated to act in order to save either the child or the embryo. However, positive obligations are contextual in a way that negative obligations are not, which means that the former are less adequate than the latter as a universal ground for minimal basic equality.

Equipped with this distinction, we can make room for the intuition that we value actualized rationality more than unactualized rationality. Consider the case above in which we had to choose between saving a conscious or an unconscious 10-year-old child. As long as we do not act deliberately against the value of life, there might be room for differentiation between human beings with and without actualized rationality, respectively. According to this line of thought, we might be justified in not promoting the 10-year-old unconscious child’s life to save the conscious child. In contrast, according to the same line of thinking, we would not be justified in using the unconscious child as a means to save the conscious child, since we would therefore be using the child merely as a means. So, to protect the value of life from such attacks, we ascribe entities with a rational nature—all human beings, including those with unactualized rationality—a right to life.

## V. RESPONDING TO REDUCTIO AD ABSURDUM ARGUMENTS AGAINST THE SUBSTANCE VIEW

Even if we acknowledge the qualifications made above about the claim that human beings have a high and equal moral value—from the very beginning—many people still believe that it is highly implausible to ascribe such a high value to an embryo. And in the literature, many objections have been raised against this claim. Many of these objections have been versions of so-called reductio ad absurdum arguments according to which a position should be rejected due to its absurd or implausible implications.

The two most common reductio arguments against the substance view are those that state that ascribing such value to an embryo implies either (a) that entities that we do not ascribe such a value to—such as ordinary cells—have this value, or (b) that we are obligated to act in an implausible way when we are forced to choose between the survival of an embryo or a born human being. My aim here is to argue against the conclusions of these reductio arguments to demonstrate the strength of the substance view in providing a basis for basic human value. It is important to notice that this strength is comparative—meaning that we must compare the strengths and weaknesses of this approach with the strengths and weaknesses of other alternative views. In applying such a comparative approach, I believe it is important to notice that the view I am defending has two very important advantages over the other views I have discussed above: this view is both binary and not implausibly exclusive. While these features speak in favor of the view I am defending here, the challenge to which I respond here is whether this view is too inclusive. I do this by arguing against the two kinds of reductio arguments raised against this view.

The first type of these reductio arguments—which we call the absurd-extension argument (Stier and Schoene-Seifert, 2013, 20)—claims that recent biotechnological advances make it at least imaginable that any cell can be converted into an entity that is sufficiently similar to an embryo. This conclusion would imply that any cell should be bestowed the same moral status as the embryo is bestowed, which would be absurd.

However, Patrick Lee defends a criterion for determining when a human organism exists that, according to him, can differentiate between human embryos and converted cells. Drawing on his definition of a human being—according to which a human organism exists “when there is a new living entity with a genetic–epigenetic state that provides the cascaded information for the development of this entity to the mature stage of a human organism” (Lee, 2011, 79). Lee argues that one can differentiate between this and other kinds of entities due to the former’s “active disposition to develop itself to the mature stage of a human organism” (2011, 79).

Critics of the substance view have questioned this claim by referring to so-called embryonic stem cells (ES cells). ES cells can be “joined to cells which are capable—[of producing] a whole, developing embryo”(Lee, Tollefsen, and George, 2014, 487). A proponent of this argument against the substance view, Jason Morris, explains the parallel in the following way.

If ES cells are added to cells that generate only placenta—then upon implantation, the ES cells will develop into a complete, viable, mature organism. However, if the transcription factors OCT4 or SOX2 are silenced in ES cells, they develop as “outer” (placenta-generating) cells rather than as embryonic tissues. (2012, 339)

Taking his point of departure from this description, he then argues that “bringing ES cells into association with cells that generate placenta is” analogous to the implantation of IVF embryos:

When IVF embryos are implanted in the uterus or when ES cells are brought together with cells that generate placenta, cells of different tissue types establish close contacts and influence each other’s development so that one cell type can provide life support for the other. In both cases, cells signal each other to alter each other’s gene expression profile and developmental trajectory. In both cases, the completion of development requires both tissue types, although the mature human being is composed of only one of the tissue types. (Morris, 2012, 341)

Consequently, Morris challenges the differentiation between embryos and other cells—like ES cells—made by proponents of the substance view, and he claims that these proponents instead need to choose: they need either to deny that the embryo has an intrinsic and active disposition to develop as a human being, or to accept that not only embryos but also other cells have this capacity, which would be absurd.

Without digging too deep into the details here, I believe it is not clear that Morris’ conclusion follows. To illustrate why this is the case, we can start by considering how this reductio argument works. According to its premises, the uterine cells alter the embryo’s genetic profile in the same way as other cells (so-called TE cells) alter the ES cells’ genetic profile; therefore, “the relationship between the uterus and the embryo is the same as the relationship between the ES cells and the TE cells” (Lee, Tollefsen, and George, 2014, 490). However, as Lee, Tollefsen, and George observe, this claim—that is, that the relationship between the uterus and the embryo is the same as the relationship between the ES cells and the TE cells—is hard to sustain, given the fact that many embryos develop without the assistance of uterine cells, which, for instance, is the case with fish and amphibians (2014, 491). Do such animals’ embryos—which can manage without uterine cells—then have a radically different disposition in nature compared to animals, such as mammals, with internal fertilization by which uterine cells assist the development of embryos? As Lee, Tollefsen, and George conclude, such a position seems quite far-stretched. They argue that it is more reasonable and economical to consider this difference between mammals on the one hand and fish and amphibians on the other as being only “with respect to nourishing and protective factors, not with respect to the fundamental genetic-epigenetic plan or instructions for maturation in the internal constitution of zygote and embryo” (Lee, Tollefsen, and George, 2014, 491). Because such a difference does not exist between ES and TE cells, this suggests a disanalogy between the relationship between these kinds of cells on the one hand and the relationship between the uterus and the embryo on the other. This in turn indicates that the embryo, in contrast to ES cells, has the “active disposition to develop itself to the mature stage of a human organism.”

I do not claim that this observation conclusively establishes the ontological status of embryos and ES cells, respectively. However, the mere doubt about the claim of the first premise—that is, that if the embryo has an active and intrinsic disposition to develop as a human being, then ES cells also have this disposition—undercuts this reductio claim since the burden of proof is on the proponents of this reductio argument to establish that x implies y. As long as this has not been done, the argument fails.

According to the other reductio ad absurdum argument, proponents of the substance view would be forced to make an implausible decision if they were forced to choose between saving an embryo or a born human being. However, as some proponents of the substance view have argued, it is compatible with the substance view to save born human beings at the expense of embryos because saving a born human being can generate more positive effects than saving embryos. The substance view and the natural-law account of morality do not exclude the possibility of making such considerations—as long as the action does not include deliberate destruction of embryos (Kaczor, 2011, 89; Friberg-Fernros, 2015). As Matthew Liao rightly notes:

…when a stranger and one’s partner are both drowning, other things being equal, one’s partner typically matters more than the stranger; but this would not show that the two have different moral status. (2010, 175)

However, the prohibition against deliberately destroying embryos—even to save human beings with actualized rationality—might generate doubts about the plausibility of this view. Consider, for example, the following situation:

A child has a deadly disease that can be cured only by using a 2-week-old human embryo. However, by using the embryo, it will be destroyed.

I assume that most people would choose to use the embryo in order to save the life of the child—even if doing so would destroy the embryo. I also assume that most people would not only make this decision but would also consider a choice not to do this to be very counterintuitive. Therefore, one might argue that this fact constitutes a reductio against the substance view. How can the proponents of the substance view respond to this charge? First, it is important to emphasize that intuitions only have presumptive epistemic authority. As the philosopher Jeff McMahan argues:

…a variety of considerations—such as the diversity of moral intuitions, the fact that people do often doubt and even repudiate certain of their intuitions, and the evident origin of some intuitions in social prejudice or self-interest—make it untenable to suppose that intuitions are infallible apprehensions of moral reality by some special faculty of moral perception. (2013, 105)

Proponents of the substance view can point to the fact that the embryo is very different from the child in many morally nonsignificant ways, such as, for instance, size, form, and/or visibility, which raises the risk that our intuitions are misguided. To illustrate, consider, for example, the following case:

A child has a deadly disease which can be cured only by the use of an early fetus. However, the only way to use the fetus is to abort it and consequently to kill it.

My claim is that we are much less certain about how we should act in this situation. Maybe some still think that it would be right to kill the fetus to save the child. However, I do not think it would be highly counterintuitive not to do it, which means that this scenario does not constitute a reductio argument against the substance view. How can we theoretically make sense of this difference between intuitions in his case and the case when the choice is between a child or an embryo? To my mind, it seems quite difficult. In fact, there are very few theories that can justify the destruction of an embryo but not of an early fetus.^9^ I believe that this conclusion should make us question the reliability of our first intuition—because it seems hard to differentiate these cases theoretically. This fact undercuts the force of this reductio argument. Maybe our strong intuitions depend on factors that are of no moral relevance—such as the size and the form of an embryo compared to what size and form we associate with a fetus.

## VI. CONCLUDING REMARKS

In this paper, I have argued that we should consider the substance view and the natural-law account of morality to respond to the problems of justifying basic equality for human beings. More specifically, I claim to have demonstrated that this approach—where human beings have basic equality due to their rational nature—has comparative advantages. It provides a binary and inclusive basis of justification for human equality. Moreover, I also claim to have demonstrated that this approach cannot be immediately rejected due to its implications. While I do not deny that some of these implications might be hard to swallow, I nevertheless believe that it is at least uncertain whether these implications constitute successful reductio arguments against this view.

## Footnotes

1 Given the fact that the claim that human beings have high and equal moral standing is widely shared, one might expect that many attempts would have been made in political philosophy to identify and defend a basis for this claim. However, this is actually not the case. For instance, Jeremy Waldron notes that while questions about equality and distributive justice have been extensively discussed on the surface level—between proponents of, for instance, egalitarianism, prioritarianism, and sufficientarianism—much less has historically been written “about the idea that we humans are fundamentally one another’s equals” (2015, 10). Likewise, Stephen Carter notes regarding the attempts to identify such a basis, that “the task of identifying this property has been strangely neglected by contemporary egalitarians” (2011, 539).

2 While I am focusing on human beings in this paper, my position certainly does not imply that no other non-human animals also might have a rational nature. I do not, however, consider that position in this paper.

3 Another way of tackling this difficulty, which I am not going to discuss in this paper, is to identify a property—such as rational agency—that, prima facie, provides a strong normative force for the claim that humans have high moral value, and then defend the equality element by arguing that differences in degrees of this property between humans do not morally matter above a certain threshold. The property in question would consequently be a threshold or range property. Proponents of this strategy include John Rawls (1999), Ian Carter (2011), and Jeremy Waldron (2015). I do not discuss this approach because I believe that we should exhaust the efforts to find a normatively significant binary property before we turn to range or threshold properties, which are scalar by nature and therefore, ceteris paribus, inferior.

4 Indeed, this seems to be Christiano’s position. According to him, “an egalitarian morality will necessarily have a kind of two-level structure: at the base is a fundamental principle such as the principles I have discussed in this chapter, but at the level of social justice we have public principles of justice that serve as shared grounds for argument and debate about how society must be structured” (Christiano, 2015, 75).

5 As I interpret Lee, he means by claiming that “having the right sort of genetic-epigenetic constitution is a necessary condition” for equal fundamental rights for *humans.* Consequently, if we encountered an alien, without our “genetic-epigenetic constitution,” but nevertheless in possession of a rational nature, then that entity would be entitled to equal fundamental rights, according to Lee’s account.

6 It is important to note that this conclusion does not imply that abortion is impermissible because abortion, according to some philosophers, is compatible with ascribing human embryos a right to life (see Thompson, 1971; Boonin, 2003). I have, however, criticized this view elsewhere (Friberg-Fernros, 2017, 37-46).

7 I also admit that there are different versions of natural-law theory; most notably, one usually distinguishes between traditional and new natural-law theory (e.g., Murphy, 2001; Lisska, 2012). In my explication of natural-law theory, I draw on the latter version, but, as far as I can see, nothing to which I commit here is incompatible with the former version.

8 This distinction of course corresponds to the distinction between negative and positive rights. As Christopher Wolfe notes, according to natural-law theory, “universally moral principles regarding particular acts are typically formulated in negative terms” (2006, 175)—even though there are some revisionist accounts of natural-law theory that seem to prioritize positive rights; see, for example, Engster (2004).

9 The only view I can come up with is Baruch Brody’s view, according to which the fetus becomes a subject of rights when its brain starts functioning, which happens in around the sixth week of pregnancy (1975, 108–9).
